# Obacunone activates the Nrf2-dependent antioxidant responses

**DOI:** 10.1007/s13238-016-0297-y

**Published:** 2016-08-16

**Authors:** Shengmei Xu, Weimin Chen, Qingfeng Xie, Yang Xu

**Affiliations:** 1 Center for Regenerative and Translational Medicine, Guangdong Provincial Academy of Chinese Medical Sciences, the Second Affiliated Hospital of Guangzhou University of Chinese Medicine, Guangzhou, 510632 China; 2 Division of Biological Sciences, University of California, San Diego, 9500 Gilman Drive, La Jolla, CA 92093 USA

**Dear Editor,**

 The transcription factor nuclear factor erythroid 2-related factor 2 (Nrf2) plays a crucial role in human antioxidant defense response against environmental insults. Small molecular chemical activators of Nrf2 can confer protection against oxidative insults and inhibit the progression of diseases related to oxidative stress. Here, we identified obacunone as a novel activator of Nrf2 by decreasing Nrf2 ubiquitination and increasing its stability. In support of these findings, the systemic administration of obacunone strongly inhibits bleomycin-induced lung fibrosis in mice. Therefore, obacunone may also provide antioxidant protection for humans against tissue damage caused by oxidative insults.

The nuclear factor-erythroid 2-related factor (Nrf2) is a redox-sensitive transcriptional factor, activity of which is tightly regulated by cytosolic protein Kelch-like ECH-associated protein 1 (Keap1) (Chen et al., 2009). The keap1-Nrf2 signaling pathway is a master antioxidant defense mechanism against environmental insults. In the absence of oxidative stress, Nrf2 is bound to Keap1 and ubiquitinated by a cul3-based E3 ligase, leading to proteasome-mediated degradation (Cullinan et al., 2004). In response to oxidative stress, the E3 ubiquitin ligase is inactivated through the modification of critical cysteine residues of Keap1, thereby allowing the accumulation of Nrf2 and its translocation into the nucleus (Zhang et al., 2004; Villeneuve et al., 2010). The activated Nrf2 exerts an intracellular antioxidant defense response through transcriptional activation of many genes including phase II detoxifying enzymes, drug transporters, antioxidant enzymes, and proteins involved in the repair and removal of damaged macromolecules (Villeneuve et al., 2010), thereby neutralizing reactive oxygen species (ROS), detoxifying harmful chemicals, and maintaining redox homeostasis. Therefore, the Nrf2 activation with small molecular chemicals can enhance body’s defense against oxidative insults.

Several small molecular chemical activators of Nrf2 have already been identified, most of which are plant-derived chemicals such as sulforaphane, tanshinone I, curcumin, BHA (Jeong et al., 2006; Nishinaka et al., 2007; Zhang and Hannink, 2003). Using *in vivo* mouse models, the Nrf2 activation induced by these activators has been shown to inhibit the progression of diseases related to oxidative stress, including diabetes, cancer, cardiovascular diseases, neurodegenerative diseases, pulmonary fibrosis, and inflammatory diseases (Chapple et al., 2012). Conversely, the Nrf2-knockout mice are prone to acute damages and tumor formation induced by various harmful chemicals (Ramos-Gomez et al., 2001; Wang et al., 2008). All these data demonstrate that Nrf2 can be used as a drug target for prevention and therapy of diseases related to oxidative stress. Therefore, the identification of novel and non-toxic Nrf2 activators is important for the development of effective dietary supplements or therapeutic drugs that protect humans against oxidative damage.

Employing the MDA-MB-231 cells stably transfected with an ARE-luciferase reporter as a screening platform (Chen et al., 2016), we identified that obacunone could induce the expression of the ARE-dependent luciferase gene in a dose-dependent manner (Fig. S1). Obacunone is a small molecular compound derived from citrus fruits with a higher content in seeds. Published studies indicate that obacunone can inhibit cancer proliferation and has some therapeutic effects on cardiovascular diseases (Poulose et al., 2006; Yoon et al., 2014). As expected, the protein levels of Nrf2 were increased in various cell types after obacunone treatment, while Keap1 levels remained constant in MDA-MB-231 cells (Fig. 1A–C). Consistent with this finding, the mRNA levels of Nrf2 target genes such as *NQO1*, *Mrp2*, and *HO-1* were increased in these cells after being treated with increasing doses of obacunone (Fig. 1D and 1E). To evaluate the impact of obacunone on Nrf2-dependent antioxidant effect, MDA-MB-231 cells were pretreated with obacunone for 8 h, and then challenged with H_2_O_2_ for an additional 12 h. Pretreatment with obacunone reduced ROS levels significantly (Fig. 1F). Therefore, obacunone can effectively protect cells from oxidative stress by activating the Nrf2 pathway.

**Figure 1:**
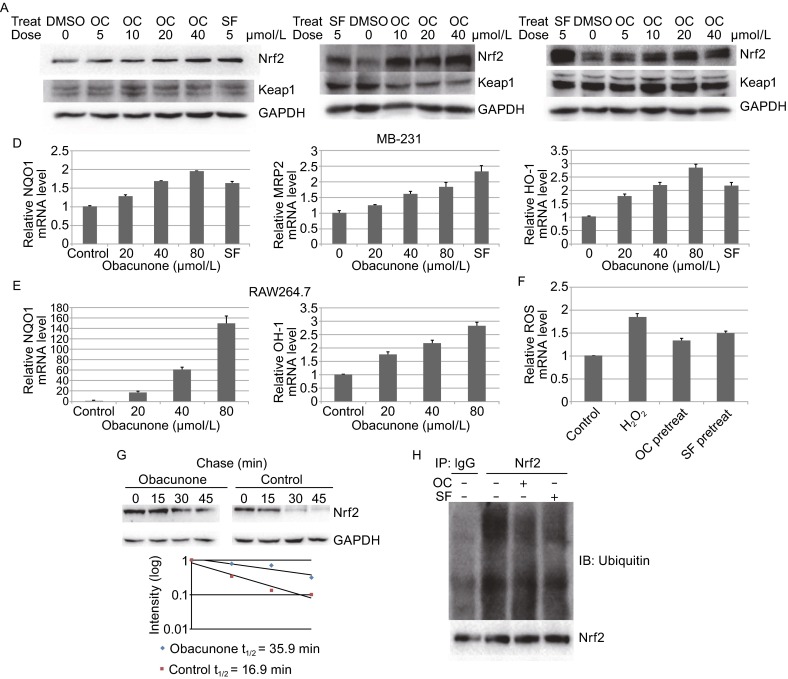
**Obacunone activates the Nrf2 pathway**. (A) MDA-MB-231 cells were treated with the indicated dose of obacunone for 4 h, and cell lysate was subject to immunoblot analysis with anti-Nrf2, -Keap1, -GAPDH antibodies. (B) RAW264.7 cells were treated with the indicated dose of obacunone for 4 h, and cell lysate was subject to immunoblot analysis. (C) LO2 cells were treated with the indicated dose of obacunone for 4 h, and cell lysate was subject to immunoblot analysis. 5 μmol/L SF treatment was included as a positive control in (A), (B), and (C). (D) MDB-MB-231 cells were either mock treated or treated with OC or SF for 24 h, then total RNAs were extracted. Relative amounts of NQO1, MRP2, and HO-1 mRNAs were measured by qRT-PCR. The standard deviations were calculated from triplicate samples. ^*^ *P* < 0.05, ^**^ *P* < 0.01 compared with its control. (E) RAW264.7 cells were either mock treated or treated with OC or SF for 24 h, then total RNAs were extracted. Relative amounts of NQO1 and HO-1 mRNAs were measured by qRT-PCR. The standard deviations were calculated from triplicate samples. ^*^ *P* < 0.05, ^**^ *P* < 0.01 compared with its control. (F) MDA-MB-231 cells were mock treated or pretreated with 80 μmol/L OC or 5 μmol/L SF for 8 h, then challenged with 0.5 mmol/L H_2_O_2_ for another 12 h. Cellular ROS level was detected by DCF staining and subsequent flow cytometry. **P < 0.01, compared with its control. ^##^ *P* < 0.01, compared with only H_2_O_2_-treated cells. (G) MDB-MB-231 cells were either mock treated or treated with 40 μmol/L OC for 4 h. 50 μmol/L cycloheximide was added and cells were lysed at the indicated time points. Cell lysates were subjected to immunoblot analysis using anti-Nrf2 and anti-GAPDH antibodies. The intensity of the bands was quantified using Quantity One software and plotted against the time after cycloheximide treatment. (H) 293T cells were co-transfected with the plasmids encoding the indicated proteins. Cells were then treated with either 5 μmol/L SF or 60 μmol/L OC along with 10 μmol/L MG132 for 4 h before cell lysates were collected for ubiquitination assay. Anti-Nrf2 immunoprecipitates were analyzed by immunoblot with anti-Nrf2 antibodies for detection of ubiquitin-conjugated Nrf2

To investigate the mechanism how obacunone increases the protein levels of Nrf2, we measured the half-life of Nrf2 protein in the absence or presence of obacunone. At the indicated time points after the addition of cycloheximide (CHX), the protein levels of Nrf2 in the absence or presence of obacunone were examined. While the half-life of Nrf2 protein was 16.9 min in the absence of obacunone, the half-life of Nrf2 protein in the presence of obacunone was increased to 35.9 min, indicating that obacunone stabilizes Nrf2 protein (Fig. 1G). To examine the impact of obacunone on the ubiquitination of Nrf2, the cells were either mock treated or treated with obacunone and the known Nrf2 activator SF. As expected, obacunone inhibits the ubiquitination of Nrf2 similarly to the positive control SF (Fig. 1H). These data support the notion that obacunone activates the Nrf2 pathway by stabilizing Nrf2 through inhibiting its ubiquitination and activating Nrf2-dependent responses.

Bleomycin is a chemotherapeutic drug used clinically for a variety of human malignancies. It has been reported that the administration of bleomycin often leads to lung injury and fibrosis in human patients. Bleomycin-induced lung injury in mice is a well-established *in vivo* model of human pulmonary fibrosis (Hoshino et al., 2009). Numerous studies have shown that the disturbance of the alveolar oxidant-antioxidant balance may play a role in the pathogenesis of chronic fibrosis (Gasse et al., 2007). Therefore, considering that the activation of Nrf2 results in antioxidant defense response, bleomycin-induced lung injury can be used to evaluate the therapeutic effects of Nrf2 activators,

We first evaluated the conditions (dose and injection frequency) that result in the activation of the Nrf2-dependent response in the lung of mice. Forty-eight hours after systemic delivery of obacunone (10 mg/kg, i.p.) in B6 mice, the protein levels of Nrf2 in the lung were upregulated (Fig. 2A). Consistent with this data, the mRNA levels of Nrf2 target genes NQO1 and HO-1 were also increased (Fig. 2B).

**Figure 2:**
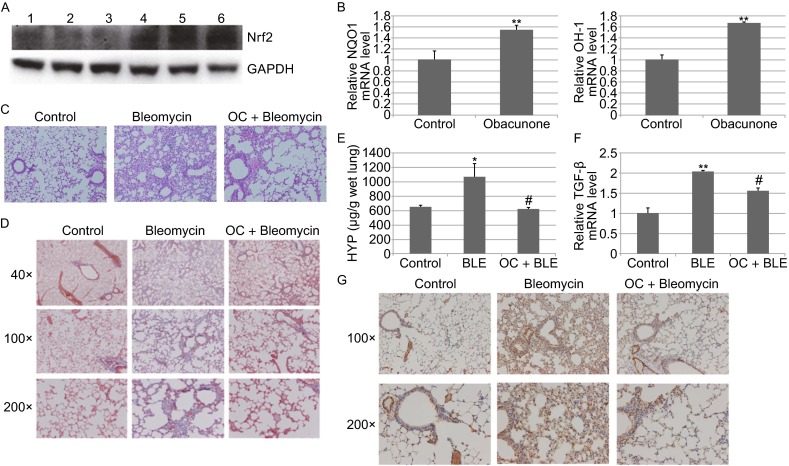
**Obacunone activates the pulmonary Nrf2 and inhibits bleomycin-induced lung fibrosis**. (A) Lung tissue lysates from mice were subjected to immunoblot analysis with anti-Nrf2, -GAPDH antibodies. Lanes 1–3 indicate control samples, lanes 4–6 indicate samples from mice subjected to OC administration. (B) Total RNAs were extracted from freshly isolated lung tissue, and relative amounts of NQO1, HO-1 mRNAs were measured by qRT-PCR. The standard deviations were calculated from triplicate samples. ^**^ *P* < 0.01, compared with its control. (C) Each group shows a representative image of the lung tissue for HE staining respectively. (D) The representative images of the lung tissue for Masson’s trichrome staining were shown. (E) HYP level in different lung tissues was compared. ^*^ *P* < 0.05, control group vs. BLM-administrated froup; ^#^ *P* <0.05, BLM-administrated group vs. OC + BLM-administrated group. (F) Relative mRNA expression of TGF-β was measured by real-time RT-PCR. ^**^ *P* < 0.01, control group compared with BLE-administrated group; ^#^ *P* < 0.05, BLM-administrated group compared with OC + BLM-administrated group. (G) Obacunone inhibits the BLM-induced lung fibrosis as revealed by IHC analysis with anti α-SMA antibodies

Bleomycin (BLM) induced lung inflammation and fibrosis (Fig. 2C). For the analysis of lung fibrosis, the collagen content in the lung tissue was examined. When compared with that in PBS-treated mice, collagen deposition (stained blue by masson’s staining) was obviously increased in the lung tissue of BLM-treated mice. Treatment of mice with obacunone significantly reduces the collagen deposition in the lung of BLM-administrated mice (Fig. 2D). To further quantify for the levels of collagen deposition, we measured the hydroxyproline content as an index of collagen, further demonstrating that obacunone treatment significantly reduces the collagen deposition in BLM-administered mice (Fig. 2E).

As additional indicators of tissue fibrosis, we examined the expression of α-SMA and TGF-β. We show that TGF-β expression was significantly upregulated in BLM-damaged lung tissue, while obacunone treatment suppressed BLM-induced TGF-β expression in the lung tissue (Fig. 2F). Consistent with this finding, the number of α-SMA+ myofibroblasts was significantly increased in the lung tissue of bleomycin-administered mice, obacunone treatment reduces the number of α-SMA+ myofibroblasts in the lung tissues of bleomycin administered mice (Fig. 2G). In summary, these findings demonstrate that obacunone can effectively protect mice against BLM-induced lung fibrosis.

BLM-induced damage causes inflammation in the lung, leading to fibrosis (Gasse et al., 2007; Hoshino et al., 2009). To understand the mechanism how obacunone protects mice against BLM-induced lung fibrosis, we examined the impact of obacunone on the BLM-induced inflammation in the lung of B6 mice. We examined the mRNA levels of several cytokines associated with pulmonary inflammation and fibrosis. In support of the notion that obacunone suppresses the BLM-induced inflammation, obacunone treatment suppressed the BLM-induced expression of inflammatory cytokines IL-6, IL-17, MCP-1, but increased the mRNA levels of IFN-γ in the lung (Fig. S2). In summary, these data support the notion that obacunone suppresses BLM-induced inflammation and fibrosis of the lung by activating Nrf2 pathway.

With accumulating knowledge of the roles of Nrf2 in protecting against environmental insults, it becomes increasingly important to identify and optimize Nrf2 activators that can protect humans from various environmental insults. We discovered a novel Nrf2 activator, a natural product obacunone. In this context, we demonstrate that obacunone can stabilize and activate Nrf2 by inhibiting Nrf2 ubiquitination. In addition, we demonstrated that oxidative stress-induced lung injury including inflammation and fibrosis could be effectively inhibited by systemic administration of obacunone. Similar to another known Nrf2 activator sulforaphane, obacunone is able to block ubiquitination and degradation of Nrf2, thus resulting in the prolonged half-life of Nrf2. It remains technically challenging to identify the direct target of obacunone in cells. Future effort will be devoted to understand the mechanism how obacunone stabilizes Nrf2. One possibility is that obacunone interferes with the function of Keap1, the E3 ligase of Nrf2, leading to the stabilization of Nrf2. Another possibility is that obacunone can disrupt the interaction between Nrf2 and Keap1, leading to the stabilization of Nrf2.

Identification of obacunone as a novel Nrf2 activator broadens the choice for new chemopreventive compounds against tissue damage or disease caused by various environmental insults. Compared with other small molecule Nrf2 activators, obacunone is a natural product with an advantage in its safety. In support of this notion, obacunone exhibits no apparent cytotoxicity *in vitro* and *in vivo* for the limited time duration tested (Fig. S3). Taken together, our experiments demonstrate the feasibility of preventing bleomycin-induced lung injury by systemic administration of obacunone, a novel potent Nrf2 activator. The obacunone-mediated intervention may also be efficacious for other types of environmental insults and confer protection against tissue damage in other organs.

## Electronic supplementary material

Below is the link to the electronic supplementary material.

Supplementary material 1 (PDF 845 kb)
