# Supplementary material for: Obacunone activates the Nrf2-dependent antioxidant responses
Source: Protein Cell. 2016 Aug 16;7(9):684–8. doi: 10.1007/s13238-016-0297-y (PMC5003787; doi:10.1007/s13238-016-0297-y)
Supplement: Supplementary file 1 — Supplementary material 1 (PDF 845 kb) [file 13238_2016_297_MOESM1_ESM.pdf]

Material and Methods

Chemicals and Cell culture

Sulforaphane (SF) was purchased from Sigma Chemical Co. (St. Louis, MO, USA). Obacunone was got from commercial chemical synthesis company.

We obtained human MDA-MB-231 breast carcinoma cells and 293T cells from American Type Culture Collection (Manassas, VA, USA). Normal hepatic cell lines (LO2) were purchased from the Nanjing KeyGen Biotech Co., Ltd. (Nanjing, China). Cells were cultured in Eagle's minimal essential medium (MEM) supplemented with 10% fetal bovine serum (FBS), 2 mM HEPES, and 6 ng/mL bovine insulin from Sigma Chemical Co.. The cells were incubated at 37°C in a humidified incubator containing 5% CO_2.

Establishment of a reporter cell line and luciferase reporter gene assay

ARE-luciferase plasmid from Donna Zhang in university of Arizona, was constructed by inserting a 39-bp antioxidant responsive element (ARE)-containing sequence from the promoter region of the human NAD(P)H quinone oxidoreductase 1 (NQO1) gene into the cloning site of the pGL4.22[luc2CP/Puro] plasmid. We transfected ARE-luciferase plasmids into MDA-MB-231 cells using lipofectamine 2000 (Invitrogen). At 48h post-transfection, cells were grown in medium containing 3 μg/mL puromycin for selection. Stable cell lines were continuously grown in the MEM containing 1 μg/mL puromycin. For the reporter gene assay, the ARE-luciferase stable reporter cells were seeded the day before and treated with different doses of test compounds for 24h. Cells were lysed, and luciferase activities were measured according to the manufacturer's instructions

(Promega). For the dual luciferase reporter gene assay, MDA-MB-231 cells were transfected with the same ARE-luciferase plasmid along with the Renilla luciferase expression plasmid, pGL4.74 [hRluc/TK], from Promega. At 18h posttransfection, the transfected cells were treated with compounds for 24h, and both firefly and renilla luciferase activities were measured with the dual luciferase reporter assay system from Promega. Firefly luciferase activity was normalized to renilla luciferase activity. The experiment was carried out in triplicate and expressed as the mean ± SD.

ROS detection

MDA-MB-231 cells were pretreated with 40 μM obacunone, 5 μM SF, or mock treated for 8 hours, then challenged with 0.5 mM H_2O_2 for additional 12 hours. Cellular ROS levels were detected by DCF staining followed by flow cytometry.

mRNA extraction and qRT-PCR

Total mRNA was extracted from cells or tissues using TRIZOL reagent (Invitrogen), and equal amounts of RNA were reverse-transcripted to cDNA using the Transcriptor First Strand cDNA synthesis Kit (Promega).

The PCR condition, as well as Taqman probes and primers for human NQO1, Mrp2 and beta-actin were reported previously [4, 15]. Briefly, we obtained the following Taqman probes from the uni-versal probe library (Roche): human Nrf2 (#70), hNQO1 (#87), hMrp2 (#73) and beta-actin (#64). The following primers were synthesized. hNrf2-forward (acacggtccacagctcatc) and reverse (tgtcaatcaaatccatgtcctg); hNQO1-forward(atgtatgacaaaggacccttcc) and reverse (tcccttgcagagagtacatgg); hMrp2-

forward(agtgaatgacatcttcacgtttg) and reverse (cttgcaaaggagatcagcaa) ; human beta-actin-forward (ccaaccgcgagaagatga) and reverse (ccagaggcgtacagggatag). The real-time PCR condition was as follows: one cycle of initial denaturation (95°C for 10 min), 40 cycles of amplification (95°C for 10 sec and 60°C for 30 sec), and a cooling period (50°C for 5 sec). The data presented are relative mRNA levels normalized to beta-actin, and the value from the untreated cells was set as 1. We used triplicate samples to determine the mean ±SD.

RT-PCR was done for mouse mRNA expression by SYBR qPCR Kit (Promega). The primer sequences for m*β*-actin , mIL-6, mIL-17, mTGF-*β*, mIFN-*γ*, mNQO1, mHO-1 are as following: mβ-actin forward (agtaccccattgaacatggc) and reverse (tcggtcaggatcttcatgag); mIL-6 forward (ctgatgctggtgacaaccac) and reverse (cagaattgccattgcacaac); mIL-17 forward (atgactcctgggaagacctcattg) and reverse (ttaggccacatggtggacaatcggtta); mTGF-*β* forward (agctgcgcttgcagagatta)and reverse (agccctgtattccgtctcct); mIFN-*γ* forward (tttctcctgcctgaaggacag) and reverse (gctcatgatttctgctctgaca); mNQO1-forward (ttctctggccgattcagagt) and reverse (ggctgcttggagcaaaatag); mHO-1-forward(cacgcatatacccgctacct) and reverse (ccagagtgttcattcgagca). The real-time PCR conditions used were the following: one cycle of initial denaturation (95℃ for 3 min), 40 cycles of amplification (95 ℃ for 15s, 60℃ for 30 s and 72℃for 20s), melting curve (95℃ for 5 s, 65℃ for 1 min and 97 ℃ continuous), and a cooling period (40 ℃ for 30 s). Mean crossing point (Cp) values and standard deviations (SD) were determined. Cp values were normalized to the respective crossing point values of the m*β*-actin reference gene. All reporter gene and RT-PCR analysis were repeated in three independent experiments and in duplicates. Data are all shown as means±SD.

Antibodies and Western blotting analysis

The antibodies for Nrf2, Keap1, and GAPDH were purchased from Santa Cruz Biotechnology (Santa Cruz, CA, USA). Cells were lysed in 1% NP-40 lysis buffer, and subjected to Western blotting. For ubiquitination assay, cells were lysed in the sample buffer [50 mM Tris-HCl (pH 6.8), 2% SDS, 10% glycerol, 100 mM DTT, 0.1% bromophenol blue]. After sonication, cell lysates were electrophoresed through an SDS-polyacrylamide gel and subjected to immunoblot analysis. For detection of the ubiquitinated Nrf2 in vitro, cells were transfected with expression vectors for hemagglutinin (HA)-ubiquitin, Keap1, and Nrf2. The transfected cells were either mock treated or treated with chemicals along with 10 μM MG132 (Sigma Chemical Co.) for 4h. Cells were lysed by boiling in a buffer containing 2% SDS, 150 mM NaCl, 10 mM Tris-HCl, and 1 mM DTT. These lysates were then diluted 5-fold in buffer lacking SDS and incubated with anti-Nrf2 antibody. Immunoprecipitated proteins were analyzed by immunoblot analysis with anti-ubqutin antibody.

Protein half-life measurement

To measure the half-life of Nrf2, cells were either left untreated or treated with 40 μM obacunone for 4 hr. To block protein synthesis, we added 50 μM cycloheximide. Total cell lysates were collected at different time points and subjected to immunoblot analysis with an anti-Nrf2 antibody. The relative intensity of bands was quantified by the ChemiDoc CRS gel documentation systemand Quantity One software from BioRad (Hercules, CA, USA).

Animals and treatments

Eight-week-old B6 mice were used for the experiment. Mice were randomly allocated into three groups (n = 6 per group): (i) corn oil; (ii) bleomycin (1mg/10g); (iii) obacunone (10 mg/kg) + bleomycin.

Obacunone and blemomycin were administrated through intraperitoneal (i.p.) injection. Briefly, B6 Mice were i.p. injected with bleomycin once every week till 3 weeks. 48 hours before bleomycin injection, mice received systemic of corn oil or obacunone (10 mg/kg). 7 days after the third injection of bleomycin, lung tissues were isolated.

Assay for hydroxyproline in lung tissues

The hydroxyproline content was determined as an index of the collagen content in the lung tissues. 300-100mg lung tissue samples were respectively hydrolyzed in 1ml lysis buffer solution (pH 7.4, 10mM Tris-HCl, 0.1mM EDTA, 10mM saccharose, 0.8%NaCl) at 100℃ for 20 min. Hydroxyproline content was then measured using the test kit (Jiancheng Bio. Company, China) according to the manufacturer’s instruction. The absorbance of colored products was measured at 550 nm.

H&E staining and Masson staining

For the histological analysis, mice were killed with an intraperitoneal injection of sodium pentobarbital (2.5-5 mg per mouse). The lungs, livers and kidneys were immediately fixed by 4% Paraformaldehyde. Sections (3.5μm thick) were cut from paraffin-embedded tissues, placed on poly-l-lysine-coated slides, and then incubated for

half an hour at 60℃. Deparaffinized sections were stained with hematoxylin and eosin (H&E) to grade the degree of alveolitis. Lung fibrosis was evaluated by Masson staining for collagen accumulation, in accordance with the manufacturers' protocol (Fuzhou Maxixin Biotech, China). The stained sections were evaluated under a light microscope.

Immunohistochemical（IHC） assay of *α*-SMA in lung tissue

The paraffin-embedded sections of lung tissue were rehydrated in xylene and graded ethanol solutions. Epitope retrieval was performed by heating sections for 3 min in 10 mM citrate buffer (pH 6.0) in a water bath at 95–100 °C in the pressure cooker. Nonspecific reactions were blocked with blocking solution of the UltraSensitive^{TM} SP （Rabbit） IHC Kit (Fuzhou Maixin Biotech, China). The sections were incubated with primary antibodies including anti-*α*-SMA antibody (1:300, Abcam, UK). After an overnight-incubation with the primary antibody at 4℃, the slides were washed three times with PBS (3 min per wash) and then incubated with the secondary antibody solution (Fuzhou Maixin Biotech, China) for 10 min at room temperature. The sections were then washed with PBS and incubated for 10 min with streptavidin–biotin peroxidase complex. After washing with PBS, *α*-SMA-positive cells were visualized using a DAB staining system (DAB Plus Kit, Fuzhou Maixin Biotech, China). Nuclei were counterstained with hematoxylin. Positive staining for *α*-SMA was brown. The stained sections were evaluated under a light microscope.

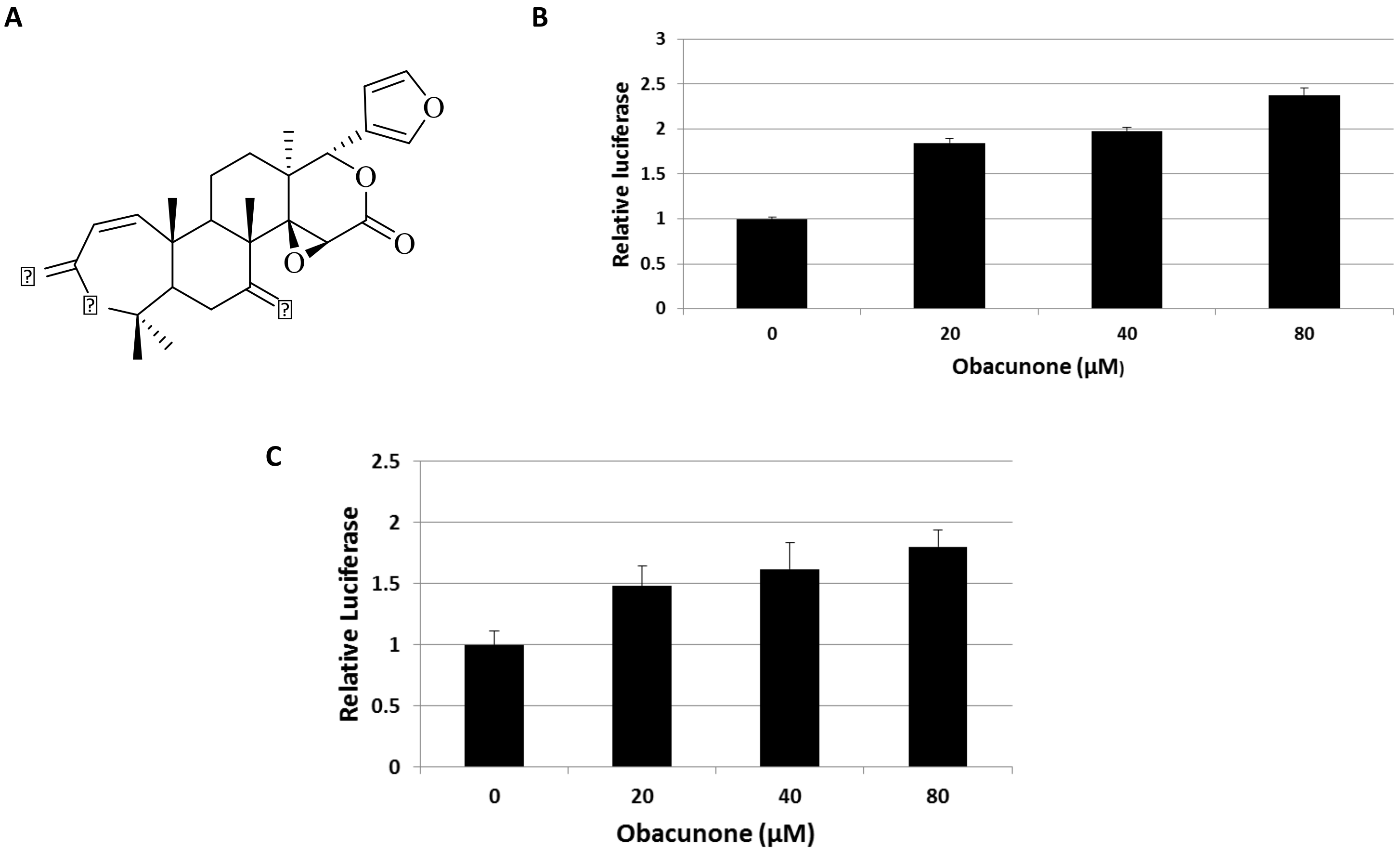

Supplementary FIG. 1. Obacunone is a small-molecule inducer of the antioxidant response element. (A) Chemical structure of obacunone. (B) Luciferase activity showing obacunone as an Nrf2 activator using a high-throughput screening system. The stable MDA-MB-231 cells expressing ARE-luciferase were seeded in 96-well plates; cells were grown to 90% confluence and treated with obacunone for 24 hours before analysis of luciferase activity. (C) Luciferase activity in MDA-MB-231 cells cotransfected with a plasmid containing a NQO1-ARE-luciferase reporter gene and a plasmid encoding renilla luciferase. The transfected cells were treated with obacunone for 24 hours prior to measurement of firefly and renilla luciferase activities in cell lysates. All luciferase reporter assays were run in triplicate and expressed as mean ± SD.

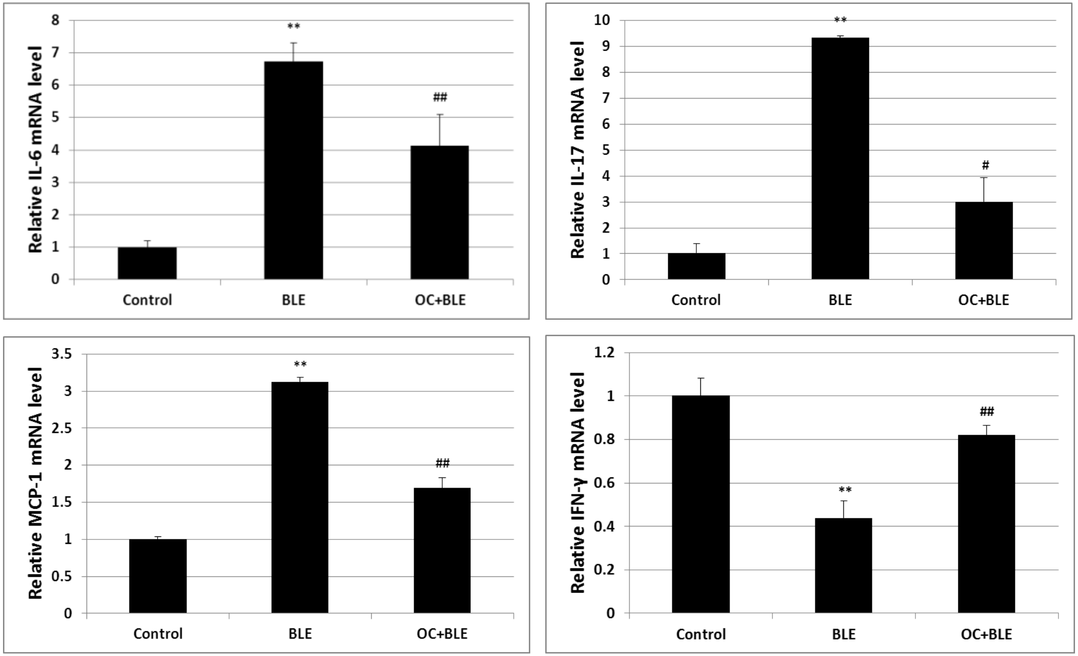

Supplementary FIG. 2. OC restores BLE-induced immune-proinflammatory cytokine production in lung tissues. Relative mRNA expression of IL-6, IL-17, IFN-γ and MCP-1 was measured by real-time RT-PCR. $^{*}p<0.05$, $^{**}p<0.01$ control group compared with BLEadministrated group; $^{\#}p < 0.05$, $^{\#\#}p<0.01$ BLM-administrated group compared with OC +BLM-administrated group.

A

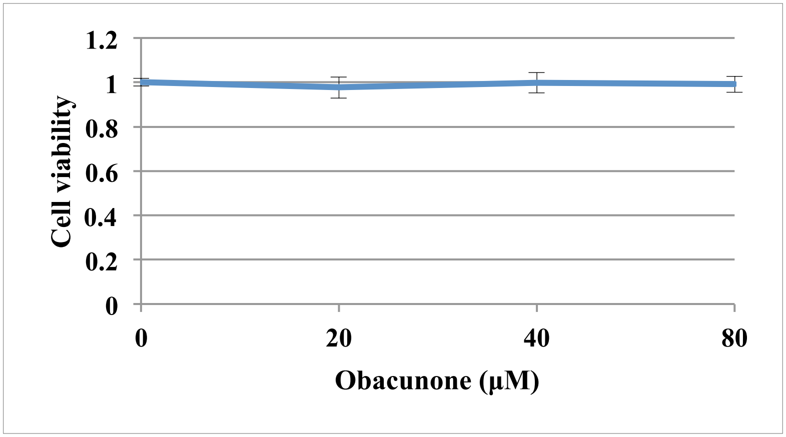

B

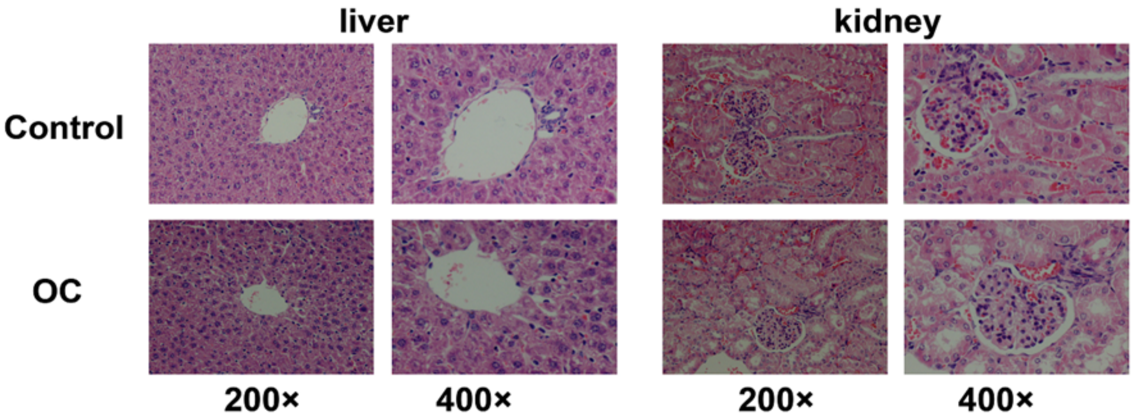

Supplementary Figure 3. (A) Cell viability was measured by MTT in RAW 264.7 macrophages treated with increasing doses of obacunone for 24h. (B) HE staining of the liver and kidney tissue shows that OC doesn't induce cytotoxicity.
